# Bryophytes in heavy metal-polluted environments: trait-based sensitivity, tolerance mechanisms, biomonitoring applications, and restoration potential

**DOI:** 10.3389/fpls.2026.1891423

**Published:** 2026-07-23

**Authors:** Wen-Zhuan Huang, Wei Huang, Lu-Zi Jin, Chang-Hong Yu, Meng-Jie Yu, Wei-Wei Chen, Yu-Huan Wu

**Affiliations:** 1 College of Life and Environmental Sciences, Hangzhou Normal University, Hangzhou, China; 2 Zhejiang Provincial Key Laboratory of Wetland Intelligent Monitoring and Ecological Restoration, Hangzhou Normal University, Hangzhou, China; 3 Zhejiang-Canada Joint Laboratory on Biodiversity and Global Change, Hangzhou Normal University, Hangzhou, China; 4 Jing Hengyi School of Education, Hangzhou Normal University, Hangzhou, China

**Keywords:** antioxidant defense, biomonitoring, bryophytes, ecological restoration, heavy metal stress, microbiome, phytochelatins, phytoremediation

## Abstract

Heavy metal contamination remains a persistent environmental challenge due to its non-degradable nature, long residence time, and cumulative ecological impacts. Bryophytes have long been recognized as effective bioindicators because of their high sensitivity and strong metal accumulation capacity. However, current research on bryophyte-metal interactions remains fragmented, with limited integration between mechanistic understanding and ecological application. In this review, we develop a trait-informed, hypothesis-generating framework that links bryophyte characteristics to heavy metal exposure, physiological responses, and ecological functions. We first examine the structural, physicochemical, and ecological attributes that govern metal interception and exposure variability, and then synthesize current evidence for extracellular immobilization, intracellular detoxification, regulatory coordination, redox buffering, and metabolic reprogramming. We further evaluate bryophyte-associated microbiomes while distinguishing functional plausibility from direct causal evidence. On the applied side, we assess bryophytes as biologically interpretable biomonitoring systems and as realistic agents of ecological stabilization and engineered biosorption. Finally, we identify key limitations, including taxonomic bias, inconsistent trait parameterization, limited experimental validation, and challenges in translating laboratory findings to field conditions. This framework currently supports directional and testable expectations rather than universal quantitative prediction, and its predictive capacity will depend on standardized cross-species experiments and field validation.

## 1 Introduction

Heavy metal contamination represents one of the most persistent and ecologically consequential outcomes of industrialization (Alloway, 2013). Unlike many organic pollutants, these elements are not biodegraded and may persist in soils and aquatic systems for decades to centuries. Their accumulation can impair plant growth, disrupt aquatic and terrestrial ecological processes, and threaten ecosystem health and biodiversity (Nagajyoti et al., 2010; Tchounwou et al., 2012). Heavy metals also pose long-term risks to human health through occupational exposure, contaminated water and food, and progressive accumulation in the body (Järup, 2003; Tchounwou et al., 2012). The problem is not a legacy issue. Mining, industrial emissions, and urban development continue to release and redistribute toxic metals across the biosphere, while historical contamination persists in many affected regions (Lu et al., 2022; Mastin et al., 2023; Zhou et al., 2023). Effective assessment and management therefore require approaches that connect metal exposure with biological response and ecological consequence. Conventional chemical monitoring is indispensable, but it cannot fully capture the organism-level and ecosystem-level meaning of metal stress without complementary biological indicators.

Bryophytes, including mosses, liverworts, and hornworts, are well suited to this role because their functional traits place them in close contact with atmospheric deposition, substrate-derived inputs, and surrounding water films. As early-diverging land plants, bryophytes provide an important evolutionary context for understanding plant adaptation to terrestrial environments (Bowman, 2022). Their value in metal-stress research, however, is determined more directly by traits such as poikilohydry, exposed gametophytic surfaces, and direct exchange of water and dissolved substances with the environment (Tyler, 1990; Varela et al., 2023). These traits make bryophytes highly sensitive to environmental perturbations, largely because many species have limited external barriers and rely on surface-mediated uptake. This sensitivity should not be viewed only as vulnerability. It also underpins their ability to intercept pollutants and register physiological responses to contamination, which explains their long-standing use in biomonitoring (Chakrabortty and Paratkar, 2006). In mine-waste and tailing systems, some bryophytes may further contribute to pioneer colonization, early surface stabilization, and revegetation, although these functions remain strongly context dependent (Ren et al., 2021; Liao et al., 2024; Liu et al., 2024).

Despite this potential, current research on bryophyte-metal interactions remains insufficiently integrated across mechanistic and applied scales. Mechanistic studies have clarified metal accumulation and tolerance in selected model or metallophyte species, whereas applied studies often emphasize accumulation patterns, biomonitoring performance, or restoration feasibility without explicitly linking these outcomes to plant traits and physiological processes (Fernández et al., 2015; Boquete et al., 2021, Boquete et al., 2022; Gezahegn et al., 2025). This separation has encouraged a second problem, namely conceptual overreach. High tissue metal accumulation is sometimes interpreted as evidence of broad remediation capacity, although accumulation, tolerance, stabilization, and extractive removal represent different biological and ecological functions. A third limitation concerns the rapid expansion of microbiome and omics studies. These approaches are valuable for discovery, but much of the current evidence remains descriptive or association-based and has not yet been translated into predictive ecological understanding (Chen and Nelson, 2022; Dangar et al., 2024; Zhu et al., 2024, Zhu et al., 2025).

To address these gaps, this review develops a trait-informed conceptual framework that links bryophyte characteristics with metal exposure, physiological response, and ecological function. The framework distinguishes three analytical components. The first comprises traits that determine metal interception and effective exposure. The second includes mechanisms that determine whether retained metals are tolerated or cause injury. The third concerns ecological functions that emerge under specific environmental conditions. This framework is not presented as a fully parameterized predictive theory. Instead, it organizes fragmented evidence, identifies sources of variation, and generates testable expectations. Greater effective surface exposure and stronger cell-wall exchange capacity are expected to enhance initial metal interception, whereas extracellular immobilization and intracellular compartmentalization may weaken the relationship between total tissue concentration and physiological damage. Similarly, life form, substrate contact, hydration history, and local adaptation are likely to shape biomonitoring signals and field persistence. By separating accumulation, tolerance, stabilization, and extractive removal, this review provides a basis for evaluating when bryophyte traits can support monitoring or restoration functions and when their application is constrained by environmental context.

## 2 Trait basis of metal exposure, sensitivity, and interpretive variation in bryophytes

### 2.1 Structural and physicochemical basis for metal interception

The high sensitivity and metal accumulation capacity of bryophytes arise partly from surface-mediated exchange between plant tissues and the surrounding environment. However, bryophytes should not be treated as structurally uniform organisms defined only by the absence of protective barriers. Their metal interception is more accurately explained by measurable traits, including exposed gametophytic surfaces, hydration regime, canopy architecture, particle retention, substrate contact, rhizoid contribution, and cell-wall exchange capacity. Many bryophytes acquire water primarily across their external surfaces and exhibit relatively limited internal control over hydration compared with vascular plants (Proctor et al., 2007). Dissolved substances and particulate metals can therefore be intercepted through exposed surfaces, although structural protection and the relative importance of different uptake pathways vary among lineages and species (García-Seoane et al., 2023; Onianwa, 2001; Varela et al., 2023). The early-diverging position of bryophytes provides evolutionary context (Bowman, 2022), whereas metal interception itself is governed mainly by functional traits that determine exposure, retention, and exchange.

Direct quantitative comparisons of cuticle thickness or surface-area-to-volume ratio across bryophytes and vascular plants remain scarce because these traits have rarely been measured across lineages using standardized protocols. Functional comparisons under shared environmental conditions nevertheless provide useful evidence for the consequences of surface-mediated interception. In an urban survey, concentrations of 12 metals in the pleurocarpous moss *Haplocladium angustifolium* were approximately 3–51 times higher than those in leaves of two co-occurring evergreen tree species, although the magnitude of this difference varied among elements and land-use types (Jiang et al., 2018). These findings support species- and context-specific evidence of enhanced metal interception by the studied moss, rather than a universal quantitative contrast between bryophytes and vascular plants.

Particulate and dissolved metals are intercepted through partly distinct processes. Particle-bound metals may be retained by sedimentation, impaction, entrapment among leaves and branches, and adhesion to rough or hydrated surfaces. These processes are influenced by growth form, canopy architecture, branching density, surface properties, particle size, rainfall wash-off, resuspension, and exposure design (Fabure et al., 2010; Fernández et al., 2015). Dissolved metals reach bryophyte surfaces through rainwater, fog, throughfall, or surface water and interact more directly with ion-exchange sites in the cell wall (Chakrabortty and Paratkar, 2006; García-Seoane et al., 2023). Bryophyte cell walls contain pectins and other polysaccharides enriched in carboxyl and other negatively charged functional groups (Popper and Fry, 2003; Krzesłowska, 2011). These groups provide binding sites for cationic metals through adsorption, ion exchange, and complexation, as supported by experimental metal–sorption studies using bryophyte materials (González and Pokrovsky, 2014). Metal retention is therefore shaped not only by external concentration but also by pH, competing ions, metal speciation, hydration–desiccation cycles, and the physicochemical state of the wall. Bryophyte surfaces should consequently be regarded as chemically active exchange interfaces rather than as inert collectors.

### 2.2 Species-, life form-, and ecotype-dependent variation in accumulation and tolerance

Shared surface–exchange characteristics do not generate uniform patterns of metal accumulation or tolerance across bryophytes. Life form can modify effective exposure by shaping shoot orientation, canopy density, branching architecture, substrate contact, water retention, and the residence time of deposited particles. Acrocarpous turfs and cushions differ from pleurocarpous mats and wefts in canopy organization and particle-interception characteristics, but these morphological contrasts do not imply a consistent direction or magnitude of metal accumulation across species and exposure conditions (Fabure et al., 2010; Stanković et al., 2021). Aquatic and semi-aquatic bryophytes may further differ from terrestrial species because sustained contact with surface water changes the relative contributions of dissolved-metal uptake, substrate-derived inputs, and ion-exchange processes (García-Seoane et al., 2023). Life form should therefore not be converted into a universal tolerance ranking. Controlled comparisons indicate that metal responses remain dependent on species identity, metal type, dose, and exposure duration, even when acrocarpous and pleurocarpous mosses are assessed under comparable experimental conditions (Sabovljević et al., 2020; Stanković et al., 2021). Experimental exposure of *Campylopus schmidii* similarly shows that metal accumulation and physiological injury vary with metal identity, exposure concentration, and treatment context (Zhang et al., 2024).

Metallophytes and moss crusts from mining environments represent an additional functional category, although they do not constitute a single morphological life form. Species such as *Scopelophila cataractae* can persist under high copper (Cu) exposure through tissue-specific and cell-wall-associated sequestration, whereas other species may accumulate comparable metal concentrations but experience rapid photochemical or growth impairment (Konno et al., 2010; Boquete et al., 2021; Printarakul and Meekinurit, 2021). Interspecific comparisons must therefore distinguish accumulation capacity from tolerance capacity. The former describes the amount of metal retained, whereas the latter requires evidence that physiological function, growth, or recovery is maintained under exposure (Boquete et al., 2021). Variation also occurs within species. Populations from contaminated habitats may exhibit greater tolerance than conspecific populations from unpolluted habitats, indicating local adaptation or ecotypic differentiation rather than a fixed species-wide response (Boquete et al., 2022).

Morphological traits, including leaf size, laminal-cell dimensions, shoot density, and canopy architecture, have been shown to covary with metal-contaminated soil conditions (Han et al., 2025). Some of these patterns may have adaptive significance. Reduced projected leaf area or a more compact canopy may lower the directly exposed surface per shoot, reduce evaporative water loss, and shield inner tissues. Variation in cell size and wall thickness may also correspond to differences in surface-area-to-volume relationships, wall-binding capacity, and water retention. Elemental concentrations have been associated with morphological variations across bryophyte species, supporting a link between tissue chemistry and structural strategy (Fernández-Martínez et al., 2021). However, reduced leaf size and restricted growth may also reflect developmental inhibition caused by toxicity. Morphological change should therefore be interpreted as adaptive plasticity only when accompanied by improved survival, recovery, or reproductive performance. Morphology alone cannot distinguish acclimation from injury.

### 2.3 Why trait context matters for interpretation

Trait variation has direct consequences for interpreting tissue metal concentrations. The same total concentration can represent different biological states depending on species identity, life form, tissue compartment, exposure pathway, hydration history, and previous selection. A tolerant population may retain a large metal load in cell walls or vacuoles while maintaining photosynthetic and growth functions, whereas a sensitive population may reach a similar concentration during progressive membrane, chloroplast, and growth impairment (Boquete et al., 2021, 2022). Total tissue concentration is therefore a composite outcome of environmental supply, interception efficiency, retention, internal distribution, and physiological handling. It cannot by itself identify either the effective dose reaching sensitive cellular targets or the degree of biological injury.

**Figure 1:**
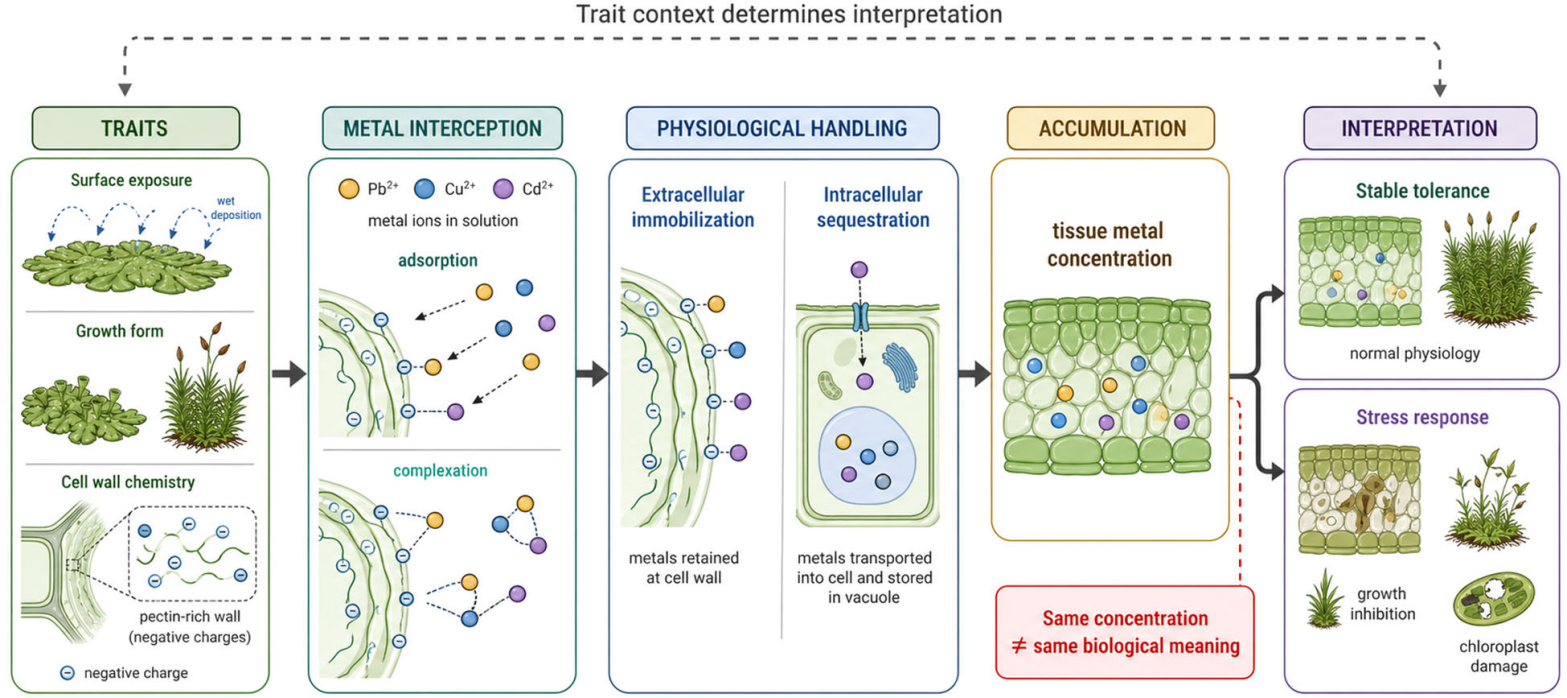
Trait-dependent framework linking metal accumulation to ecological interpretation in bryophytes. Metal accumulation in bryophytes reflects a sequence of processes from trait-mediated interception to physiological handling. Structural and physicochemical traits influence metal retention, internal distribution, and detoxification, so similar tissue concentrations may represent different biological states. Trait context is therefore essential for interpreting biomonitoring signals and linking accumulation data to ecological meaning.

This context dependence does not weaken bryophyte biomonitoring, but it defines the conditions required for reliable standardization. Within a monitoring network, the use of the same species, comparable shoot segments and tissue ages, standardized washing and digestion procedures, and consistent exposure durations can reduce trait-related variation (Ares et al., 2012; Fernández et al., 2015; Capozzi et al., 2016, Capozzi et al., 2017). When multiple species are used, species effects should be calibrated rather than ignored. The same principle applies to remediation. High tissue concentration should not be equated automatically with effective removal because it may reflect stable extracellular sorption, intracellular accumulation, low biomass, or incipient toxicity. Trait context therefore links concentration, mechanism, and application. It explains why bryophytes should not be treated as interchangeable biomonitoring samplers or as functionally uniform candidates for remediation. Structural and physiological differences determine both metal retention patterns and their ecological meaning (**Figure 1**).

## 3 The plant-intrinsic mechanisms of heavy metal tolerance in bryophytes

**Figure 2:**
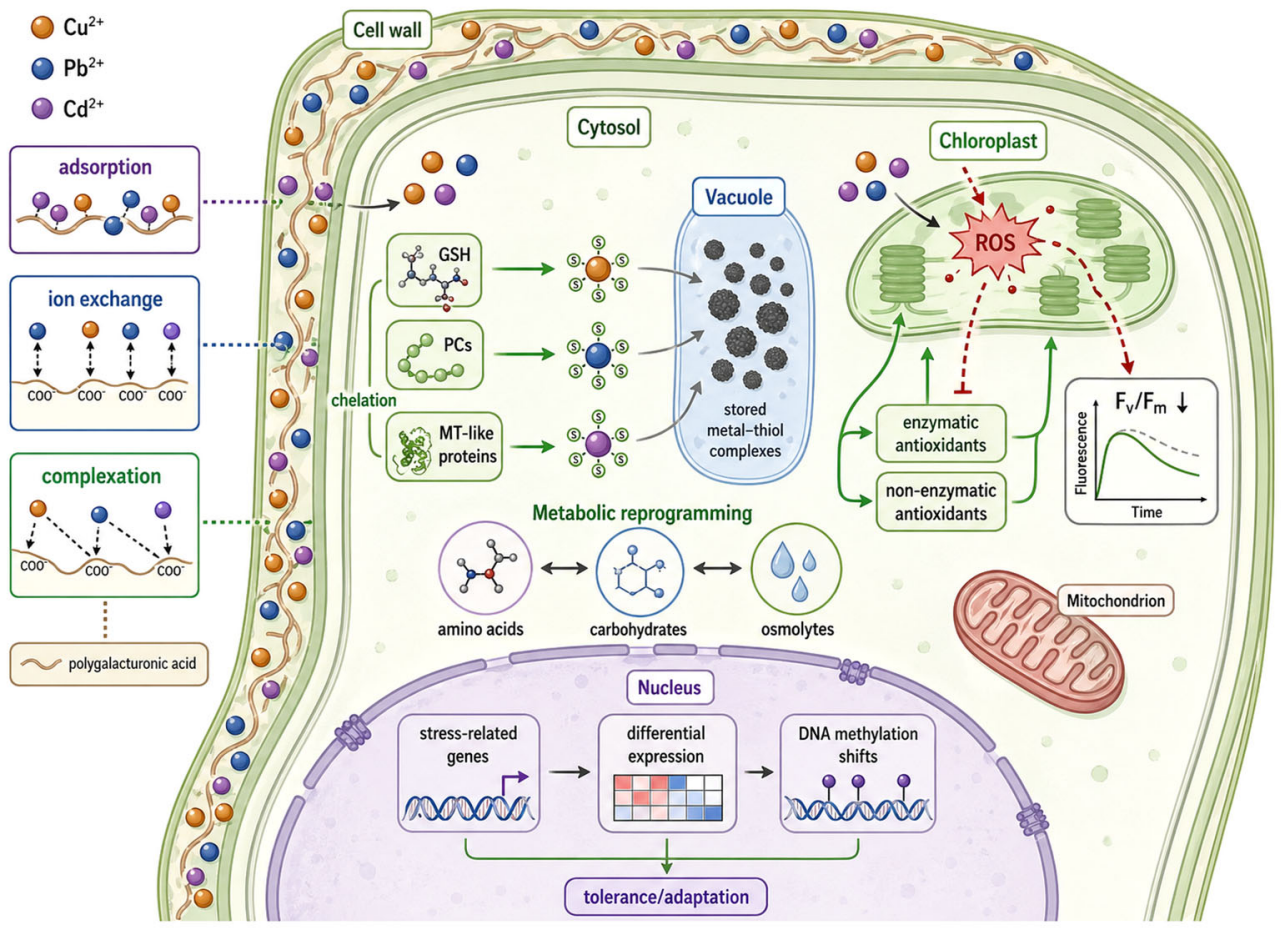
Multi-layered mechanisms of heavy metal tolerance in bryophytes. Heavy metal tolerance in bryophytes involves coordinated processes across extracellular, intracellular, and regulatory levels. Metals can be intercepted by cell-wall components through adsorption, ion exchange, and complexation. After entry into cells, detoxification may involve thiol-mediated chelation, vacuolar sequestration, antioxidant defense, and metabolic adjustment. These processes jointly influence metal distribution, cellular injury, and physiological performance.

Bryophyte tolerance to heavy metals is generated by interacting processes that operate across cellular compartments, regulatory networks, and physiological functions. For analytical clarity, these processes can be considered at three connected levels. The first involves spatial control of metal availability through extracellular immobilization and intracellular sequestration. The second involves regulatory coordination of transport, homeostasis, redox balance, and protective pathways. The third involves functional acclimation of photosynthesis, antioxidant systems, and central metabolism. This organization should not be interpreted as a fixed chronological cascade. Metal sensing, transcriptional regulation, metabolic adjustment, and redox feedback may overlap, interact bidirectionally, and vary in importance among species, metals, doses, and exposure durations (**Figure 2**).

### 3.1 Spatial control of metal availability through extracellular immobilization and intracellular sequestration

A recurring feature of bryophyte tolerance is the spatial control of metal retention. Cell walls constitute a major extracellular compartment, binding metals through adsorption, ion exchange, complexation, and localized precipitation. In *Scopelophila cataractae*, Cu is strongly associated with protonemal wall pectins, while lead (Pb) deposits in the walls of *Funaria hygrometrica* protonemata can subsequently be remobilized (Konno et al., 2010; Krzesłowska et al., 2010; Itouga et al., 2017). These observations indicate that extracellular retention can reduce immediate metal access to sensitive intracellular targets, but they also show that wall-bound metals are not necessarily immobilized permanently. Evidence from *Campylopus schmidii* further supports this interpretation. Under simulated acid-rain conditions, this moss restricted Cu and cadmium (Cd) migration, suggesting that moss tissues and associated surface structures can function as local metal-retention interfaces rather than passive accumulators alone (Zhang et al., 2025).

Stress-induced cell-wall remodeling may further modify this buffering capacity. In plants more broadly, trace-metal exposure can modify wall-polysaccharide composition, wall thickness, pectin abundance, and the proportion of low-methyl-esterified pectins. Pectin de-esterification increases the availability of carboxyl groups and may enhance the density of negatively charged binding sites for cationic metals (Krzesłowska, 2011). In bryophytes, Cu localization in wall pectins and the contribution of extracellular Cd detoxification are consistent with the hypothesis that pectin chemistry contributes to metal binding (Konno et al., 2010; Bellini et al., 2023). However, these findings do not by themselves demonstrate stress-induced pectin de-esterification. Direct measurements of metal-induced changes in pectin methyl-esterification remain limited to a small number of taxa and experimental systems. Cell-wall remodeling should therefore be treated as a plausible inducible defense supported by localization and biochemical evidence, but not yet as a universal response across bryophyte lineages.

Once metals move beyond the extracellular barrier, intracellular compartmentalization provides a second layer of detoxification, a mechanism widely recognized across plants (Hall, 2002; Clemens, 2006). Ultrastructural and cytochemical studies show that bryophytes can sequester metals in vacuoles, sulfur-rich deposits, and other intracellular sites, thereby reducing direct interaction with chloroplasts, membranes, and enzyme systems (Carginale et al., 2004; Basile et al., 2012; Maresca et al., 2022). Thiol-based detoxification is central to this process. Glutathione can contribute directly to redox buffering and also serves as a precursor for phytochelatin synthesis. Phytochelatins then bind cytosolic metal ions and facilitate sequestration into less sensitive compartments (Cobbett and Goldsbrough, 2002; Bellini et al., 2020, Bellini et al., 2023; Salbitani et al., 2023). This dual role is especially evident in liverwort systems. In *Marchantia polymorpha*, glutathione and phytochelatins jointly support both intracellular and extracellular Cd detoxification (Bellini et al., 2023). In *Lunularia cruciata*, a Cd/Fe/Zn-responsive phytochelatin synthase points to an ancient and functionally meaningful chelation machinery in early land plants (Degola et al., 2014).

Mosses show related but not identical detoxification patterns. In *Leptodictyum riparium*, severe Cd stress induces glutathione transferase and phytochelatin synthase activity, yet phytochelatins do not appear to represent the only or consistently dominant detoxification route (Bellini et al., 2020; Maresca et al., 2022). Metallothionein-like genes add another component to the metal-binding toolkit. In *Physcomitrium patens*, several such genes respond differentially to stress, and available functional evidence suggests partial redundancy and metal-specific roles rather than a simple one-gene, one-function model (Pakdee et al., 2019). The balance between extracellular immobilization and intracellular handling therefore varies with species identity, habitat, and metal context. Aquatic and terrestrial bryophytes may process metals differently, and patterns observed in one model system should not be generalized without comparative validation (Basile et al., 2012; Suzuki et al., 2016; Bačkor et al., 2023; García-Seoane et al., 2023). Nevertheless, a common mechanistic principle emerges. Bryophyte tolerance depends strongly on where metals are retained and how securely they are bound.

### 3.2 Regulatory coordination and omics-supported candidate pathways

Regulatory responses coordinate metal transport, chelation, redox homeostasis, protein protection, and metabolic adjustment. Comparisons between tolerant and sensitive populations of *Scopelophila cataractae* have identified constitutive and metal-responsive differences in genes associated with ion transport, metal homeostasis, reactive oxygen species (ROS) detoxification, and cellular protection. These differences are associated with contrasting tolerance phenotypes and suggest that contamination history may contribute to distinct regulatory states (Boquete et al., 2022). However, the causal roles of individual genes and pathways remain to be experimentally established. Functional analyses of metallothionein-like genes in *Physcomitrium patens* likewise indicate metal-specific functions and partial redundancy, rather than a simple relationship between a single gene and tolerance (Pakdee et al., 2019). These studies identify regulatory components consistent with tolerance, but the strength of inference depends on whether candidate pathways are functionally validated.

Transcriptomic and untargeted metabolomic data should primarily be interpreted as hypothesis-generating evidence. Differentially expressed genes, altered metabolites, and enriched pathways can reveal biological processes that covary with metal exposure or tolerance phenotypes. They cannot, by themselves, establish whether these responses are upstream drivers of tolerance, downstream consequences of injury, or components of a general stress response. In *Tortella tortuosa*, Cd exposure was associated with shifts in metabolite profiles and pathways related to amino-acid and carbohydrate metabolism, together with changes in antioxidant status and phyllosphere bacterial composition (Zhu et al., 2024, Zhu et al., 2025). These coordinated responses broaden the range of candidate mechanisms, but they do not distinguish causal tolerance mechanisms from secondary responses to Cd-induced damage. Such distinctions require targeted validation through genetic manipulation, biochemical inhibition or complementation, subcellular localization, dose–response analysis, and recovery assays. The current evidence therefore supports a distinction between direct functional evidence, mechanistic association, and omics-based correlation.

Epigenetic regulation may provide an additional layer linking environmental history to persistent regulatory states. Patterns observed in tolerant and sensitive populations of *Scopelophila cataractae* are compatible with a possible epigenetic contribution to long-term metal responses beyond transient transcriptional activation (Boquete et al., 2022). However, these patterns may also covary with genetic differentiation, habitat history, or other environmental factors. Demonstrating an epigenetic contribution to tolerance will require common-garden or multigenerational experiments, targeted manipulation of candidate epigenetic modifications, and evidence that altered epigenetic states change metal-response phenotypes independently of underlying genetic variation.

### 3.3 Functional acclimation through photosynthetic adjustment, redox buffering, and metabolic reprogramming

The photosynthetic apparatus is both a sensitive target of metal toxicity and a useful endpoint for evaluating physiological effects. Heavy metals can impair chlorophyll stability, photosystem II performance, electron transport, and redox balance in plants, but bryophyte-specific inference should rely primarily on experiments conducted in mosses and liverworts rather than on broad extrapolation from vascular plants. In *Leptodictyum riparium*, heavy-metal exposure alters photosynthetic yield, glutathione-related metabolism, phytochelatin synthesis, and the cellular localization of thiol-rich compounds (Bellini et al., 2020; Maresca et al., 2022). These responses show that photosynthetic performance should be interpreted together with detoxification and redox regulation. Similarly, Cu exposure in *Taxiphyllum barbieri* affects chlorophyll content, chlorophyll fluorescence, lipid peroxidation, and antioxidant status, supporting the use of photosynthetic indicators as informative but incomplete markers of tolerance or injury (Bačkor et al., 2023). Chlorophyll fluorescence parameters are therefore most reliable when combined with pigment content, oxidative-damage markers, membrane integrity, growth, and recovery after stress removal (Baker, 2008; Chen et al., 2015, Chen et al., 2019). Therefore, antioxidant activity should be interpreted together with ROS or H_2_O_2_ accumulation, MDA/TBARS or other membrane-injury indicators, chlorophyll-fluorescence parameters such as Fv/Fm, ΦPSII, and NPQ, pigment content, growth or biomass production, and recovery after stress removal.

Metabolic reprogramming extends beyond changes in individual antioxidant enzymes. Studies of Cd-exposed *Tortella tortuosa* have reported coordinated shifts in amino-acid and carbohydrate metabolism, osmolyte-related pathways, secondary metabolites, and phyllosphere bacterial composition (Zhu et al., 2024, Zhu et al., 2025). These patterns indicate system-level physiological and host-associated responses to Cd exposure and identify processes that may contribute to acclimation or tolerance. However, their causal roles remain unresolved without targeted functional validation. Comparable metabolomic changes reported under water-deficit and nitrogen treatments or during dehydration-rehydration cycles should be interpreted more cautiously. Because these studies did not involve heavy-metal exposure, they provide evidence for potentially overlapping general stress-response modules rather than direct evidence of metal tolerance (Liu et al., 2016; Yang et al., 2023). Metabolomic evidence should therefore be treated as hypothesis-generating unless candidate metabolites are validated by targeted biochemical assays, flux analysis, inhibition, complementation, or recovery experiments.

## 4 Beyond the host: bryophyte-associated microbiomes and the holobiont perspective

### 4.1 Microbiome restructuring under metal stress

**Figure 3:**
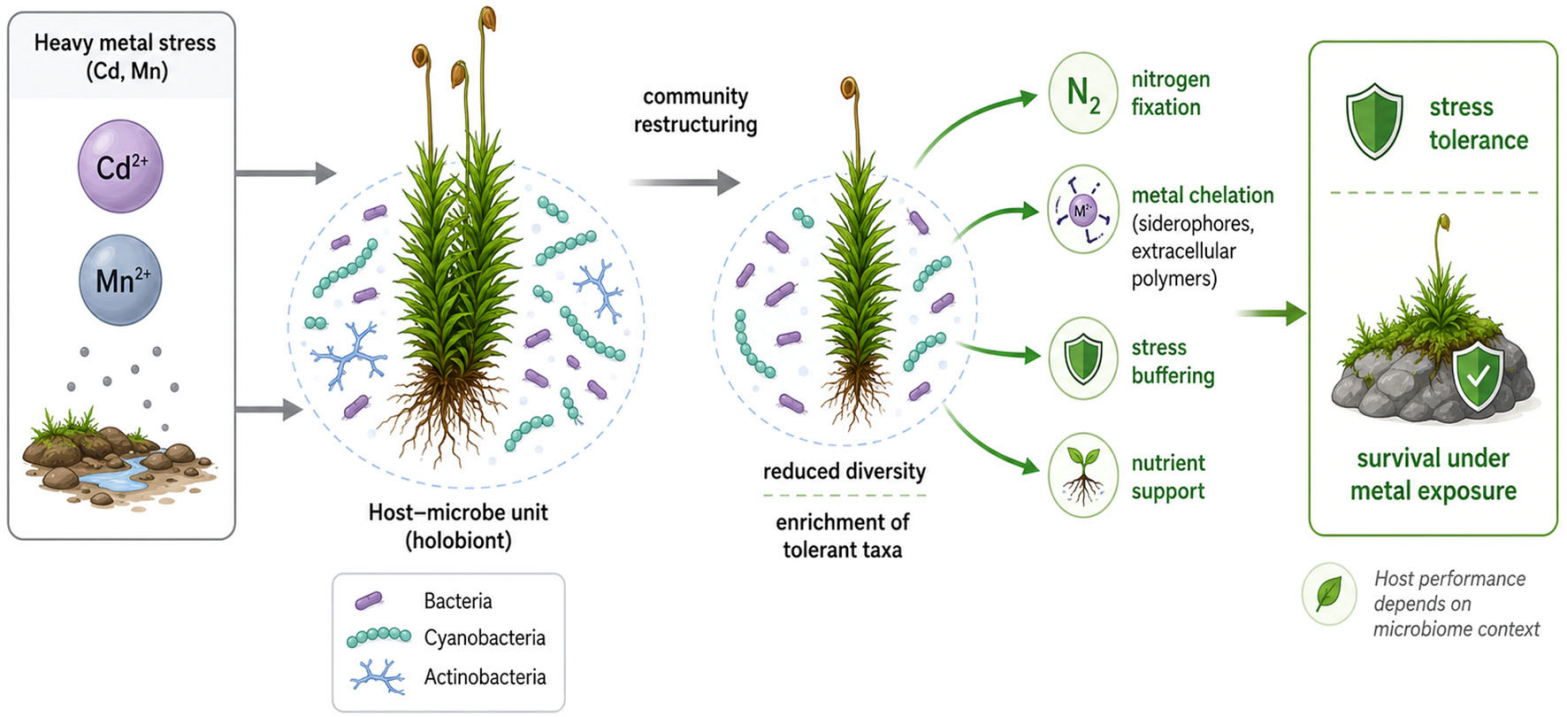
Bryophyte-associated microbiomes under heavy metal stress. Bryophytes host diverse microbial communities that may respond to metal contamination through changes in composition and potential function. Associated microorganisms may influence nutrient supply, metal availability, oxidative–stress responses, and local microenvironmental conditions. Current evidence supports a plausible plant–microbe contribution to bryophyte performance under metal stress, although causal roles require direct experimental validation.

Heavy metal contamination can reshape microbial communities in bryophyte-dominated systems through interacting environmental and host-associated filters (**Figure 3**). Metal concentration is important, but community responses also depend on metal identity, dose, oxidation state, speciation, and bioavailability. Environmental conditions, including pH, moisture, redox status, organic matter, and nutrient availability, can influence both metal toxicity and microbial niche suitability. Host species, growth form, surface chemistry, hydration status, and stress-related metabolic changes may impose additional filters, while contamination duration, season, successional stage, and prior exposure provide historical context for community assembly.

Available studies support contamination-related microbial restructuring across several bryophyte-associated compartments, although these compartments are not always defined consistently. Field investigations in manganese (Mn)-mining areas have reported pollution- and succession-related shifts in soil bacterial communities beneath moss cover (Chen et al., 2023), as well as changes in moss-associated and rhizosphere-associated bacterial assemblages (Ma et al., 2024). Experimental Cd exposure has also been associated with concurrent changes in bryophyte physiology, metabolite profiles, and phyllosphere bacterial communities (Zhu et al., 2024). Comparative evidence under simulated Cd gradients further indicates that *Mnium laevinerve*, *Timmia bavarica*, and *Brachythecium salebrosum* exhibited species-specific patterns of Cd accumulation, physiological response, metabolite variation, and phyllosphere bacterial restructuring (Lin et al., 2026). Together, these studies indicate that microbial responses are jointly associated with contaminant dose and host identity. However, they remain primarily association-based and do not demonstrate that microbiome shifts causally enhance host Cd tolerance. Microbiome restructuring should therefore be interpreted as the combined outcome of direct metal toxicity, environmentally mediated metal availability, host physiological state, and species-specific host filtering.

Metal contamination may also alter functions already performed by resident microbial communities (**Figure 3**). Under comparatively undisturbed conditions, bryophyte-associated microorganisms have been implicated in nitrogen fixation, methane oxidation, nutrient mobilization, organic-matter transformation, and other biogeochemical processes, although the strength of evidence differs among functions and bryophyte systems (Chen and Nelson, 2022). In *Sphagnum*, experimental evidence shows that methane-oxidizing symbionts can assimilate methane-derived carbon and transfer part of this carbon to the photosynthetic host (Raghoebarsing et al., 2005). Under metal stress, sensitive nitrogen-fixing, decomposer, or mutualistic taxa may decline, functional redundancy may be reduced, and microbial resources may shift toward efflux, chelation, oxidative-stress defense, and cellular repair. Consistent with this possibility, nitrogen fixation associated with co-occurring mosses responds differently to Cu and zinc (Zn) contamination, suggesting that host identity and community composition mediate functional sensitivity (Sjøgren et al., 2023). Enrichment of metal-resistant taxa should therefore not be interpreted as functional improvement. A microbial community may become more resistant to contamination while losing part of its nutrient-cycling or host-supporting capacity.

### 4.2 Potential microbial contributions to host performance under metal stress

Once metal contamination has reconfigured bryophyte-associated microbial assemblages, the more critical question is whether these changes alter host performance. Current evidence does not yet justify treating microbial effects as established tolerance mechanisms. A more defensible interpretation is that associated microorganisms represent a set of testable functional hypotheses. They may support bryophytes through nutrient acquisition, nitrogen fixation, phosphate mobilization, extracellular polymeric substance production, siderophore release, phytohormonal signaling, oxidative stress buffering, and local microenvironment modification (Bragina et al., 2013; Dangar et al., 2024; Mishra et al., 2025). Evidence from broader plant microbiome research shows that microorganisms can alter metal availability and host stress responses through siderophore-mediated metal binding, nutrient mobilization, and microbial signals that promote growth (Rajkumar et al., 2010). In bryophytes, however, these pathways remain more credible as mechanistic possibilities than as proven determinants of metal tolerance.

Evidence involving defined bryophyte-associated isolates illustrates both the value and the limits of current inference. Inoculation with the actinobacterium *Micromonospora chalcea* CMU55–4 improved growth-related traits of *Physcomitrium sphaericum*, including carotenoid content, fresh and dry mass, capsule production, and acclimatization performance. The isolate also showed genomic potential for siderophore production, phosphate solubilization, compatible-solute metabolism, and oxidative-stress responses (Insuk et al., 2020). This study demonstrates that a bryophyte-associated bacterium can modify host performance. Because the experiment was not conducted under heavy metal exposure, it should not be cited as direct evidence for microbial enhancement of metal tolerance. Studies that link Cd exposure with changes in the phyllosphere microbiome and metabolome of *Tortella tortuosa*, or Cu and Zn contamination with altered moss-associated nitrogen fixation, provide stronger evidence within metal contexts (Sjøgren et al., 2023; Zhu et al., 2024). Even in these cases, causality requires controlled microbiome manipulation.

Potential benefits should also be weighed against neutral or negative outcomes. Severe contamination may remove beneficial and metal-sensitive microorganisms simultaneously, and taxa enriched because of intrinsic metal resistance are not necessarily mutualists (Hassani et al., 2018; Chen et al., 2023; Ma et al., 2024; Zhu et al., 2024). Microorganisms may compete with bryophytes for limiting nitrogen, phosphorus, or labile carbon. They may also increase metal exposure when acidification, siderophore release, or redox transformation raises metal solubility (Rajkumar et al., 2010; Gadd, 2010). Declines in microbial diversity and functional redundancy may further reduce the resilience of the host-microbiome system to subsequent drought, warming, or nutrient disturbance (Allison and Martiny, 2008; Bragina et al., 2013; Slate et al., 2024). Thus, enrichment of metal-resistant microorganisms should not be read automatically as improved host performance.

Broader studies of bryophyte microbiomes provide additional ecological precedent for functional coupling between hosts and associated microorganisms. In *Sphagnum* systems, microbial partners participate in carbon- and nitrogen-related processes, and methane-oxidizing symbionts can transfer methane-derived carbon to the photosynthetic host (Raghoebarsing et al., 2005; Chen and Nelson, 2022). In pollution-affected peatland or recovery settings, bryophyte-associated microbial communities may also remain functionally relevant or show patterns consistent with ecological resilience and recovery, although these observations do not demonstrate direct microbial control of host performance (Seward et al., 2023). They nevertheless support a useful premise for metal stress research. Bryophyte performance may depend not only on host cell wall binding, chelation, and redox regulation, but also on the functional state of associated microbial communities.

### 4.3 Value and limits of the holobiont framework

The holobiont framework is most useful when it is treated not as a claim of universal microbial benefit, but as a way to place bryophyte metal responses within a broader biological interface. For bryophytes, this interface is unusually exposed. Poikilohydry, direct surface exchange, and close contact with substrates create continuous opportunities for microbial colonization and environmental interaction (Chen and Nelson, 2022; Dangar et al., 2024; Mishra et al., 2025). From this perspective, metal tolerance is not only a property of host cells, cell walls, or detoxification pathways. It may also be influenced by the microbial assemblages that occupy bryophyte surfaces and surrounding microhabitats. The conceptual value of the holobiont view therefore lies in expanding the scale of interpretation while preserving the central role of host traits, metal chemistry, and habitat context (Bordenstein and Theis, 2015; Vandenkoornhuyse et al., 2015).

This framework is particularly relevant in contaminated environments because bryophyte performance often varies among sites that appear similar in total metal burden. Current studies show that bryophytes harbor diverse and potentially functional microbial communities, and that metal exposure is associated with shifts in microbial assemblages across soil beneath moss cover, moss-associated compartments, rhizosphere-related habitats, and phyllosphere communities (Chen et al., 2023; Ma et al., 2024; Zhu et al., 2024). These patterns suggest that metal stress reshapes not only bryophyte physiology but also the biological context in which that physiology operates. Host identity, surface chemistry, hydration regime, substrate condition, and contamination history may jointly determine which microbial partners persist and which functions remain available under stress. The holobiont perspective therefore helps explain why tissue-level mechanisms alone may be insufficient to account for field variation in bryophyte tolerance, persistence, and recovery (Dangar et al., 2024; Mishra et al., 2025).

The strength of this framework, however, depends on careful separation between explanation and evidence. Most metal-context microbiome studies still report associations among contamination, microbial composition, host physiology, metabolite profiles, and environmental gradients rather than experimentally demonstrated mechanisms (Sjøgren et al., 2023; Zhu et al., 2024; Lin et al., 2026). Such evidence is valuable because it identifies candidate interactions and reveals ecological structure, but it cannot determine whether microbial shifts enhance host tolerance, passively track host injury, or result from direct metal filtering of microbial taxa. Evidence from pollution-affected peatlands may indicate microbial stability or recovery-related patterns, yet these observations do not prove that microbial communities control bryophyte persistence under metal stress (Seward et al., 2023). The holobiont framework should therefore be used as a hypothesis-generating lens rather than as evidence that associated microorganisms generally improve bryophyte metal tolerance (Bordenstein and Theis, 2015; Queller and Strassmann, 2016).

A balanced use of the holobiont framework requires a clear transition from association to mechanism and, ultimately, to application. The framework is valuable because it expands the explanatory unit from the bryophyte host alone to the host and its associated microbiota, but it should not be used to infer microbial benefit without functional evidence (Bordenstein and Theis, 2015; Vandenkoornhuyse et al., 2015; Queller and Strassmann, 2016). Association-based studies can identify microbial groups, predicted functions, or community states that covary with metal exposure, as shown by studies linking contamination with moss-associated nitrogen fixation, phyllosphere microbiome restructuring, metabolomic shifts, or peatland microbial recovery patterns (Seward et al., 2023; Sjøgren et al., 2023; Zhu et al., 2024; Lin et al., 2026). These studies provide a necessary basis for hypothesis generation, but they cannot determine whether microbial assemblages reduce metal toxicity, simply reflect host stress, or intensify exposure. Future work should therefore prioritize axenic or microbiome-reduced bryophytes, reciprocal inoculation, synthetic microbial communities, microbiome transplantation, and field-linked mesocosms that measure metal localization, redox injury, photosynthetic performance, growth, survival, recovery, and ecological persistence. Only through such manipulation-based evidence can the holobiont perspective move from an informative ecological lens to a predictive framework for interpreting and applying bryophyte responses in contaminated environments.

## 5 Bryophytes as ecologically informative indicators for environmental monitoring

### 5.1 Why bryophytes work as biomonitors

The value of bryophytes in environmental monitoring emerges directly from their functional traits. Exposed surfaces, recurrent hydration-desiccation cycles, and wall-mediated metal binding collectively allow their tissues to integrate atmospheric deposition and local environmental inputs over time (Onianwa, 2001; Chakrabortty and Paratkar, 2006; Aboal et al., 2010). These traits help explain why moss surveys have been widely used to reconstruct regional patterns of metal deposition. However, the strength of bryophyte biomonitoring lies not only in elemental accumulation, but also in the ecological interpretation of that accumulation. Growth form, canopy structure, substrate contact, and hydration dynamics can influence particle retention and the relative contribution of atmospheric and substrate-derived metals. The magnitude and direction of these effects require species- and design-specific calibration (Fabure et al., 2010; García-Seoane et al., 2023). Thus, bryophyte biomonitoring should be understood as a trait-informed indicator system in which organisms integrate spatial and temporal environmental variability rather than function as passive sampling materials (Markert et al., 2003).

### 5.2 Passive and active biomonitoring as complementary approaches

**Figure 4:**
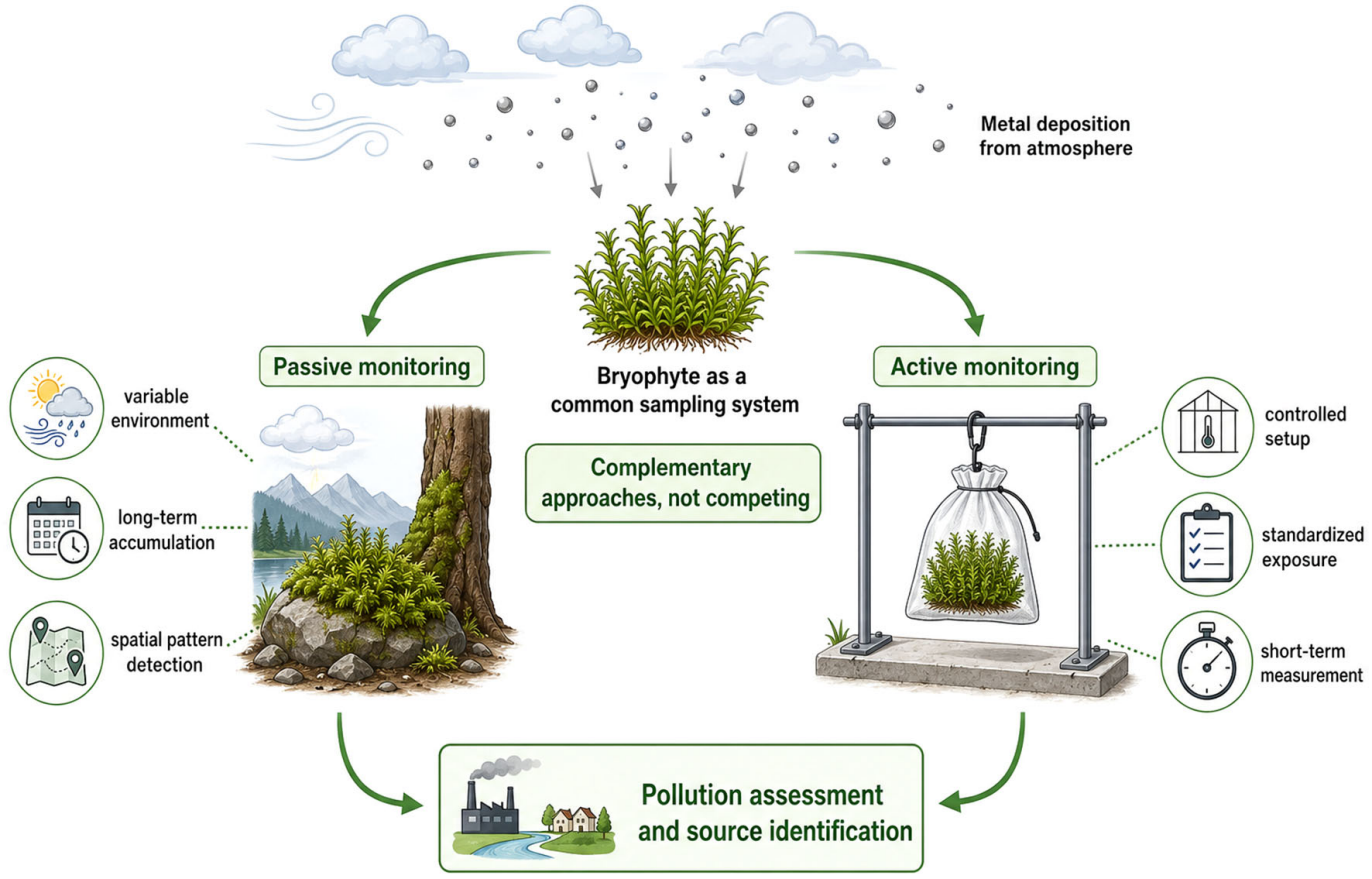
Passive and active biomonitoring approaches using bryophytes. Bryophyte-based biomonitoring includes two complementary strategies. Passive monitoring uses native bryophytes to reflect long-term environmental conditions, whereas active monitoring employs transplanted moss bags under standardized exposure. These approaches differ in experimental control and ecological integration, but both can provide useful information for assessing pollution patterns, temporal trends, and potential sources.

In field applications, bryophyte-based biomonitoring typically employs two complementary strategies, passive monitoring with native populations and active monitoring with transplanted moss bags (Ares et al., 2012), as illustrated in **Figure 4**. Passive monitoring is particularly useful for reconstructing spatial gradients, identifying long−term deposition patterns, and comparing present contamination with historical archives (Harmens et al., 2010, Harmens et al., 2015). Its trade−off is limited control over plant age, background contamination, and microhabitat variability. Active monitoring, conversely, delivers rigorous experimental control. By standardizing species identity, initial elemental background, and exposure duration, active monitoring is particularly valuable where native bryophytes are absent or where short-term deposition needs sharper resolution (Capozzi et al., 2016, Capozzi et al., 2017). These approaches therefore differ in inferential strength rather than methodological quality. Passive monitoring emphasizes ecological integration, whereas active monitoring emphasizes controlled exposure.

Representative case studies illustrate this complementarity clearly. Seasonal moss-bag deployment in Xichang revealed higher overall metal burdens in spring, pronounced pollution near a major motorway, and substantial anthropogenic source contributions (Mao et al., 2022). In Moscow, roadside *Sphagnum* bags detected elevated antimony (Sb), uranium (U), thorium (Th), and other elements, helping discriminate anthropogenic from geogenic inputs in urban recreational areas (Shvetsova et al., 2024). Passive monitoring, meanwhile, has been particularly effective for resolving broad regional deposition patterns and cumulative contamination signals over longer periods. European moss surveys have documented long-term declines in many metal concentrations, providing evidence of temporal changes consistent with shifts in emissions and atmospheric deposition (Harmens et al., 2010, Harmens et al., 2015). Regional studies in China have primarily resolved spatial contamination patterns and probable source contributions rather than directly evaluated the effectiveness of emission-control policies (Zhou et al., 2017, Zhou et al., 2023). Accordingly, method selection must align strictly with the inferential goal. Passive monitoring is better suited to ecological integration and long-term spatial assessment, whereas active monitoring is better suited to controlled exposure, source apportionment, and short-term resolution.

### 5.3 From large-scale surveys to source apportionment

The European moss survey network, particularly the International Cooperative Program on Effects of Air Pollution on Natural Vegetation and Crops (ICP Vegetation), remains one of the strongest demonstrations of bryophyte biomonitoring at large spatial scales. Since 1990, five-year surveys have produced long-term datasets that support analysis of temporal trends, spatial deposition patterns, and changes consistent with emission reductions and policy shifts (Harmens et al., 2010, 2015). National and regional studies extend this approach across different environmental settings. In Albania, Qarri et al. (2019) surveyed *Hypnum cupressiforme* at 55 sites and found concentrations of aluminum (Al), iron (Fe), chromium (Cr), nickel (Ni), and vanadium (V) above European baseline values, suggesting combined geological and industrial influences. In Georgia, the first nationwide moss survey established baseline concentrations for 41 elements, with enrichment-factor analysis indicating mixed natural and anthropogenic sources for Th and zirconium (Zr) (Chaligava et al., 2021). In Bulgaria, comparisons between the 2005/2006 and 2015/2016 surveys revealed declines in Pb, Cd, and Zn that coincided with major changes in industrial activity, while increases in several crustal elements were consistent with greater contributions from transport and road construction (Hristozova et al., 2020).

Other national and regional studies demonstrate the breadth of bryophyte-based monitoring. In Canada, moss elemental profiles were used to characterize spatial contamination patterns and distinguish probable natural and anthropogenic source contributions (Goodarzi et al., 2001). In Italy, moss-bag comparisons across urban, suburban, and rural settings revealed spatial differences in atmospheric contamination and supported discrimination among land-use and pollution contexts (Capozzi et al., 2016). In Turkey, bryophyte surveys were likewise used to identify areas of elevated elemental accumulation and evaluate spatial patterns and potential pollution sources (Coskun et al., 2011). In China, the approach has expanded rapidly in recent years. Studies using native *Haplocladium microphyllum* in Wuxi, Taizhou, and Huai’an showed that bryophyte chemistry can be integrated with multivariate analysis to support source apportionment, distinguishing industrial emissions, coal combustion, traffic, agriculture, and mining (Yan et al., 2016; Zhou et al., 2017, 2023). Positive matrix factorization in Huai’an further resolved six major contributing sources, demonstrating that bryophytes do not just map pollution; they help unmix it (Zhou et al., 2023). These findings highlight the capacity of bryophyte biomonitoring for both pollution assessment and source apportionment at regional scales.

### 5.4 Standardization, species choice, and trait−informed interpretation

Bryophyte biomonitoring is reliable only when biological and methodological variation are explicitly controlled. Species selection must be trait-informed and regionally justified because canopy architecture, surface roughness, hydration dynamics, substrate contact, and tissue chemistry influence pollutant retention and signal stability (Fernández et al., 2015; García-Seoane et al., 2023; Varela et al., 2023). A persistent limitation in early biomonitoring was the implicit treatment of bryophyte species as interchangeable sampling materials. This assumption is no longer tenable. Established monitoring networks account for species identity, regional differences, sampling design, and analytical protocols rather than assuming universal equivalence among moss taxa (Harmens et al., 2015; Zhou et al., 2017). When results from multiple species must be combined, interspecies calibration should be applied only to species-element combinations for which sufficiently robust relationships have been demonstrated (Galsomiès et al., 2003; Carballeira et al., 2008).

Species-specific accumulation patterns may strengthen bioindicator value, but they should not be converted automatically into universal tolerance rankings. *Scopelophila cataractae* is strongly associated with Cu-enriched substrates and can localize Cu in specific tissues and cell-wall-associated compartments, a distribution pattern consistent with reduced exposure of photosynthetically active tissues (Boquete et al., 2021; Printarakul and Meekinurit, 2021). Moss crusts dominated by *Ditrichum brevidens*, *Ditrichum flexicaule*, and *Oxystegus cylindricus* in bauxite tailing areas also differ in elemental accumulation patterns and potential value as indicators of soil contamination. Among these crust types, *Ditrichum brevidens* showed comparatively high accumulation of Al, Mg, Ca, Fe, K, and Ti (Wang et al., 2025). *Bryum argenteum* has been reported from multi-metal-contaminated Mn slag sites and shows substantial metal uptake, supporting its occurrence and accumulation capacity under those field conditions rather than establishing broad species-wide tolerance (Lu et al., 2022). Such cases demonstrate bioaccumulation and bioindicator potential, but physiological tolerance requires additional evidence from survival, growth, photosynthetic performance, recovery, or reproductive output under controlled exposure.

Community-level patterns can provide additional information when interpreted within appropriate ecological limits. In heavy metal-contaminated grasslands in Poland, variation in bryophyte composition, species richness, and abundance was evaluated as an indicator of soil contamination and habitat conditions (Rola and Plášek, 2022). This supports community-level bioindication, but it does not by itself demonstrate a direct role in restoration or successional facilitation. Tissue metal concentration should also be matched to the intended inference. For atmospheric biomonitoring, concentration or enrichment is primarily an index of integrated exposure and deposition, not a stand-alone measure of physiological injury. When physiological effects are the focus, elemental analysis should be combined with chlorophyll fluorescence, pigment content, oxidative-damage markers, visible injury, growth, or recovery. The fact that an identical tissue concentration may represent different biological states does not undermine bryophyte biomonitoring when biological material and inferential objectives are standardized. It mainly cautions against uncalibrated comparisons among species, protocols, and monitoring contexts.

## 6 Bryophytes in remediation and ecological restoration: realistic functions and boundaries

### 6.1 Pioneer establishment and ecological stabilization in contaminated habitats

Bryophytes may contribute to restoration primarily through pioneer establishment and ecological stabilization rather than through direct contaminant removal. They can colonize some nutrient-poor, erosion-prone, and metal-enriched substrates, including sites where vascular-plant establishment is limited (Kong et al., 2023; Qiao et al., 2025). In such settings, their ecological value lies less in rapid biomass production than in their potential to protect exposed substrates, retain moisture, reduce erosion, moderate near-surface microclimatic conditions, and contribute to the gradual development of biologically active surface layers. Functional studies of bryophyte and moss-crust systems support roles in moisture retention, erosion mitigation, and microclimatic modification, whereas observations from post-mining habitats indicate their relevance for early colonization and surface-layer development (Širka et al., 2018; Ren et al., 2021).

This stabilizing role may be particularly important in physically degraded landscapes, including mine tailings, waste-rock surfaces, spoil heaps, and steep exposed slopes. By covering exposed surfaces, retaining moisture, trapping fine particles, and contributing to early organic-matter accumulation, bryophytes and bryophyte-dominated crusts can modify near-surface conditions and improve substrate stability (Kong et al., 2023; Liao et al., 2024; Qiao et al., 2025). Experimental evidence from a Pb–Zn tailing pond shows that induced moss biocrusts can increase soil moisture and water-holding capacity and reduce the exchangeable fraction of Pb (Liao et al., 2024). In pyritic tailings, a naturally occurring plant blanket containing mosses and herbaceous plants reduced water infiltration and the downward migration of Cd and Cu under precipitation-leaching conditions, although these effects cannot be attributed to bryophytes alone (Lin et al., 2024). These findings support a role for bryophyte-containing surface covers in local stabilization, but they do not yet establish broad restoration success.

Evidence from post-mining systems illustrates several distinct aspects of bryophyte establishment and ecological relevance. Field investigations in Canada and Slovakia document bryophyte occurrence and vegetation patterns across disturbed substrates and successional settings, but these observations do not by themselves demonstrate that bryophytes actively improve soil conditions or drive succession (Širka et al., 2018; Liu et al., 2024). In China, moss-community composition has been examined in relation to Fe, Mn, Cd, and sulfur contamination in a Mn-mining area (Han et al., 2022), while cultivation experiments have evaluated the feasibility of establishing bryophytes on rare-earth tailings (Shen et al., 2022). Studies beneath moss-dominated biocrusts have additionally identified relationships between moss cover and the structure and potential functions of associated soil microbial communities (Kong et al., 2023). Collectively, these studies support bryophyte colonization, community-level responses, cultivation feasibility, and associations with soil microbial processes in mining environments. Whether such changes promote early succession should be tested directly using substrate properties, vascular-plant recruitment, seedling survival, vegetation-cover development, and community turnover.

### 6.2 Realistic remediation pathways versus overstated phytoextraction expectations

Bryophytes exhibit strong metal-sorption and accumulation capacities, as demonstrated in laboratory sorption studies and field investigations of metal-contaminated habitats (González and Pokrovsky, 2014; Lu et al., 2022). Reviews have also proposed several potential operational advantages for bryophyte-based remediation, including relatively low maintenance requirements and the capacity to function under stressful conditions, although these advantages dependent on species identity, deployment method, and field context (Gezahegn et al., 2025). However, treating bryophytes as generic phytoremediation candidates can obscure important functional boundaries. Current evidence supports several distinct application pathways, including *in situ* stabilization, early-stage ecological buffering, and engineered biosorption. These pathways should be distinguished from large-scale phytoextraction because the traits that favor surface stabilization, pioneer establishment, or sorption do not necessarily support substantial removal of contaminant stocks.

*In situ* stabilization is supported by studies showing that moss biocrusts or plant covers containing bryophytes can improve water retention, modify metal mobility, and reduce water infiltration or the downward migration of metals in contaminated substrates, although the contribution of bryophytes must be distinguished from that of co-occurring vascular plants (Liao et al., 2024; Lin et al., 2024). Early-stage ecological buffering is supported by evidence that bryophytes can establish on disturbed mine substrates, contribute to surface cover, and participate in the early recovery of degraded habitats (Ren et al., 2021; Liu et al., 2024). Engineered biosorption represents a separate application in which harvested or processed bryophyte biomass is used as a sorbent under controlled treatment conditions (González and Pokrovsky, 2014; Tesser et al., 2021). Conflating living bryophyte covers with biomass-based sorption can overstate ecological remediation capacity by translating engineering adsorption performance into field-scale restoration claims.

The distinction between tissue concentration and total removable metal mass is central to evaluating bryophyte-based remediation. Some bryophytes exhibit high tissue metal concentrations, but concentration alone does not demonstrate effective removal of contaminant stocks. In general phytoextraction theory, removal capacity depends on tissue concentration, harvestable biomass, repeated biomass removal, and the fraction of the contaminant pool that can be accessed (Pulford and Watson, 2003). Direct bryophyte-specific estimates of harvestable metal removal per unit ground area remain scarce. One field study in a moss-dominated alpine ecosystem estimated long-term accumulation rates of approximately 5.6 mg Pb m^−2^ yr^−1^, 0.4 mg Cd m^−2^ yr^−1^, and 27.6 μg Hg m^−2^ yr^−1^ (Wang et al., 2019). These values indicate accumulation and retention within a moss–organic soil system rather than contaminant removal through harvesting. They should therefore not be interpreted as evidence of ecosystem-scale phytoextraction. Similarly, substantial metal uptake by *Bryum argenteum* and other colonizing plants in Mn-slag fields demonstrates accumulation and local retention under field conditions, but not extraction efficiency at the ecosystem scale (Lu et al., 2022).

**Figure 5:**
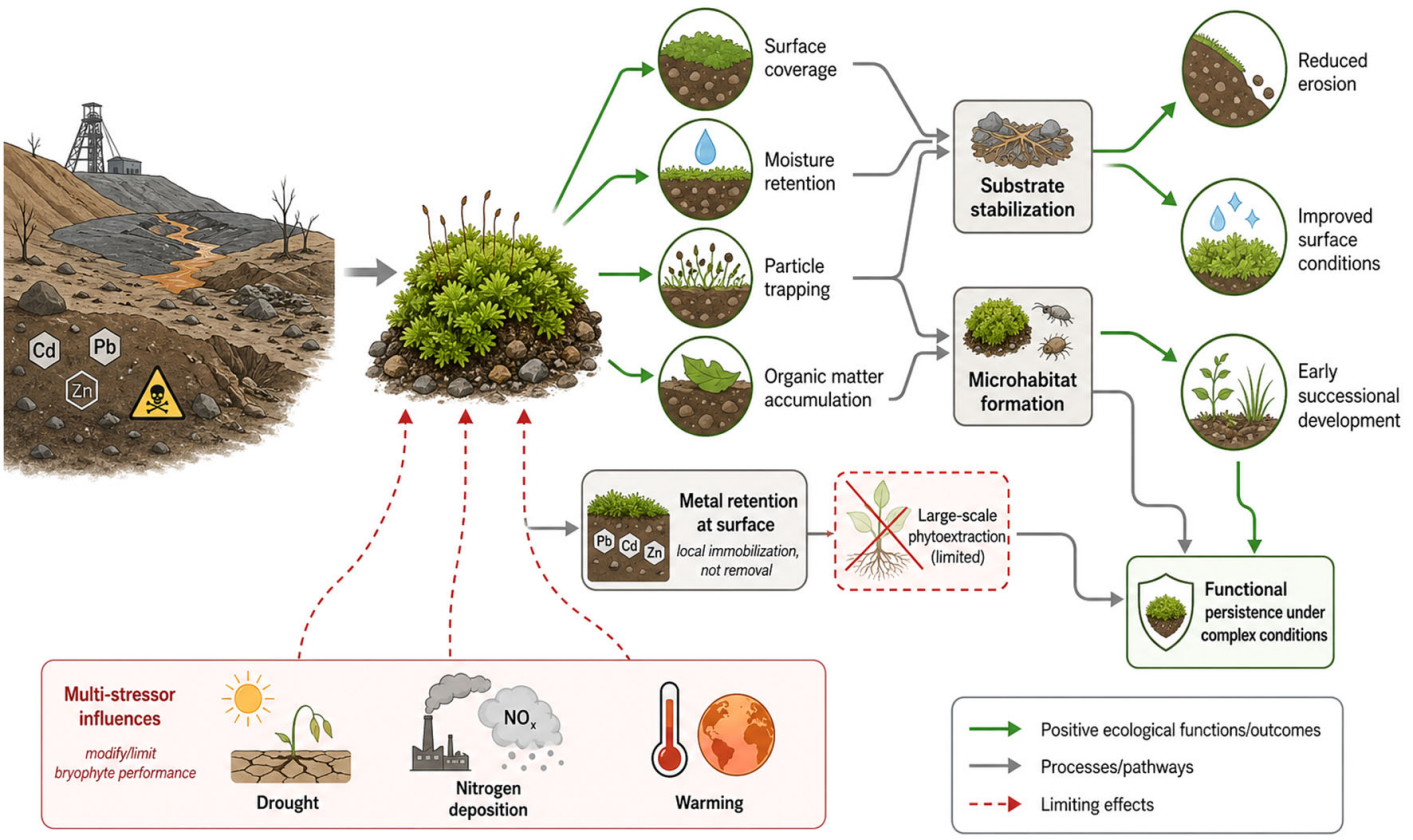
Ecological functions and application boundaries of bryophytes in contaminated environments. Bryophytes can contribute to environmental assessment and management as indicators of metal deposition, pioneer colonizers that stabilize exposed substrates, and biomass-based biosorbents in engineered systems. Their contribution to large-scale phytoextraction is constrained by biomass production, harvestability, contaminant accessibility, and field persistence. These functions are further influenced by substrate conditions, metal mixtures, climate-related stressors, and associated microbial communities.

High tissue concentration may result from stable wall adsorption, intracellular sequestration, low biomass, progressive toxicity, or a combination of these processes. It should not be used alone to rank remediation performance. For living bryophyte covers, relevant indicators include metal retention per unit ground area, biomass production, survival, cover persistence, erosion reduction, metal remobilization after desiccation or senescence, and the maintenance of facilitative functions under field stress. For harvested biomass used as an engineered sorbent, more appropriate criteria include adsorption capacity, selectivity, regeneration, disposal, and the stability of retained metals (González and Pokrovsky, 2014; Tesser et al., 2021). Moss bags efficiently accumulate airborne metals under standardized exposure conditions and are useful for active biomonitoring, but they should not be equated with engineered metal-removal systems (Ares et al., 2012; Shvetsova et al., 2024). Current restoration evidence also remains geographically concentrated, particularly in mining systems from China and a limited number of post-industrial sites elsewhere. Bryophytes should therefore be explicitly positioned within the remediation toolbox primarily as stabilizers and early-stage facilitators, rather than as agents of large-scale phytoextraction (**Figure 5**).

### 6.3 Multi-stressor constraints on field performance and restoration outcomes

A major challenge in translating bryophyte-based restoration from experimental promise to field application is that contaminated environments are influenced by multiple interacting stressors rather than by metal exposure alone. In natural and semi-natural systems, bryophytes may simultaneously experience drought, nitrogen deposition, warming, substrate instability, and changes in associated microbial communities. Existing studies indicate that such co-stressors can independently or jointly alter bryophyte physiological performance, community structure, and ecological function, although their effects on metal availability and metal tolerance remain context dependent and have not been demonstrated for all stressor combinations (Liu et al., 2016; Ma et al., 2024; Slate et al., 2024).

Nitrogen deposition provides a useful example of this complexity. Depending on species identity and ecological strategy, nitrogen inputs may either alleviate or exacerbate heavy metal stress. In the nitrogen-demanding moss *Plagiomnium acutum*, combined Pb and nitrogen exposure enhances chlorophyll content, osmolyte accumulation, and antioxidant enzyme activity relative to Pb stress alone, indicating partial alleviation of toxicity under the tested conditions (Wang et al., 2024). In the same study, *Syntrichia caninervis* and *Bryum argenteum* exhibited physiological injury under nitrogen treatment alone, whereas combined Pb–nitrogen exposure produced slightly less severe responses than nitrogen treatment alone, as indicated by reduced membrane lipid peroxidation and increased osmolyte contents and antioxidant enzyme activities (Wang et al., 2024). These treatment-specific responses should not be interpreted as fixed species-level nitrogen-sensitivity traits.

This complexity matters directly for restoration. A bryophyte that tolerates a given metal under controlled conditions may fail to maintain its stabilizing, buffering, or facilitative functions in the field when additional abiotic and biotic pressures reduce its physiological resilience. Studies of drought, warming, and related environmental pressures suggest that climate-associated co-stressors may alter bryophyte hydration dynamics, carbon balance, and microbial interactions, thereby modifying the ecological context in which metal stress occurs (Liu et al., 2016; Slate et al., 2024). Because these studies do not directly test metal availability, they should be interpreted as supporting a climate–metal co-stressor hypothesis rather than as evidence that climate pressures necessarily change metal bioavailability. Bryophyte-based restoration should therefore be evaluated not only by short-term physiological tolerance, but also by functional persistence under realistic field pressures. Relevant outcomes include sustained surface cover, substrate stabilization, moisture regulation, metal mobility, vascular-plant recruitment, seedling survival, and community development (Ma et al., 2024; Qiao et al., 2025). Short- to medium-term field and mesocosm studies provide initial evidence for induced moss-biocrust establishment in Pb–Zn tailings, reduced water infiltration and Cd and Cu migration beneath plant blankets containing mosses and herbaceous plants, nutrient and seedling responses in waste-rock mesocosms, and improved Jack pine establishment in bryophyte-treated mining-polluted rocky outcrops (Liao et al., 2024; Lin et al., 2024; Malo et al., 2025; Gery et al., 2026). Multi-year field experiments and long-term monitoring that incorporate seasonal variability, interacting stressors, substrate heterogeneity, and microbial-community dynamics are still required to determine whether these effects persist at restoration-relevant scales.

## 7 Conclusions and future perspectives

### 7.1 Integrated tolerance strategies

Bryophytes exhibit a coordinated tolerance system that operates across structural, physiological, molecular, and ecological levels under metal stress. At the environmental interface, cell-wall polysaccharides containing carboxyl and other negatively charged functional groups provide potential binding sites for metal ions (Popper and Fry, 2003; Krzesłowska, 2011; Stanković et al., 2018). Direct evidence from Cu localization in wall pectins, experimental metal sorption analyses, and metal-migration studies supports the role of bryophyte surfaces and cell walls in extracellular metal retention, which may limit or delay metal entry into metabolically sensitive cellular compartments (Konno et al., 2010; González and Pokrovsky, 2014; Zhang et al., 2025). Once internalized, metals may be detoxified through glutathione- and phytochelatin-mediated chelation and sequestered into intracellular compartments such as vacuoles, thereby reducing cytotoxicity (Stanković et al., 2018; Maresca et al., 2022; Salbitani et al., 2023). These processes are coupled with antioxidant defenses, where enzymatic and non-enzymatic systems act in concert to mitigate oxidative damage (De Agostini et al., 2022; Salbitani et al., 2023).

Tolerance also involves broader physiological and regulatory adjustment. Osmotic regulation, metabolic reprogramming, and persistent regulatory or potentially epigenetic variation may contribute to cellular stability under fluctuating stress conditions (Boquete et al., 2022; Zhu et al., 2024, 2025). Emerging evidence further suggests that these responses may extend beyond the host plant. Variation in associated microbiomes is linked to nutrient availability, metal-related processes, and host stress responses, consistent with a possible plant–microbe contribution to performance in contaminated environments (Chen et al., 2023; Dangar et al., 2024; Ma et al., 2024). However, most microbiome evidence remains associative, and causal roles require direct manipulation. Overall, bryophyte tolerance should be interpreted as an integrated but context-dependent outcome of exposure, retention, detoxification, physiological buffering, and ecological interaction.

### 7.2 Key research gaps

Despite substantial progress, several limitations constrain current understanding of bryophyte responses to metal contamination. A central limitation is the uneven strength of inference across evidence types. Direct causal evidence includes genetic disruption or complementation, targeted inhibition, controlled removal and reintroduction of microorganisms, and experiments showing that manipulation of a candidate mechanism changes tolerance or recovery. Subcellular localization, biochemical assays, dose-response relationships, and recovery experiments provide intermediate mechanistic support. Transcriptomic, metabolomic, epigenetic, and community-composition studies are indispensable for discovery, but they primarily identify associations and candidate pathways. They cannot alone distinguish an upstream tolerance mechanism from a downstream injury response or a correlated environmental effect (Boquete et al., 2022; Zhu et al., 2024, 2025). Conclusions in this field should therefore be explicitly matched to the level of evidence on which they are based.

Taxonomic and ecological coverage remains another major limitation. Detailed mechanistic studies are still concentrated in a relatively small set of mosses and metallophytes, including *Lunularia cruciata*, *Leptodictyum riparium*, *Scopelophila cataractae*, *Taxiphyllum barbieri*, *Marchantia polymorpha*, and *Campylopus schmidii* (Degola et al., 2014; Bellini et al., 2020; Boquete et al., 2021, 2022; Bačkor et al., 2023; Bellini et al., 2023; Zhang et al., 2024). Liverworts, hornworts, non-model taxa, and many species used in biomonitoring remain insufficiently represented. This imbalance limits the extent to which tolerance mechanisms can be generalized across evolutionary lineages, growth forms, and habitat types. It also weakens the predictive value of trait-based frameworks when they are applied beyond the few taxa for which detailed physiological and molecular evidence is available.

A further gap lies between controlled experiments and field performance. Many studies still rely on single metal treatments, stable hydration regimes, and short exposure periods, whereas bryophytes in contaminated habitats experience metal mixtures, fluctuating water availability, nitrogen deposition, temperature variation, substrate heterogeneity, and microbiome change. Field and mesocosm studies from mining and tailing systems show that bryophyte performance is shaped by substrate conditions, co-occurring vegetation, microbial communities, and multiple environmental stressors (Liu et al., 2016; Liao et al., 2024; Lin et al., 2024; Ma et al., 2024; Slate et al., 2024). Laboratory tolerance should therefore not be treated as a direct proxy for long-term ecological persistence. The key unresolved question is which traits and mechanisms remain robust when metal stress is embedded within realistic environmental complexity.

The functional interpretation of metal accumulation also requires greater precision. Accumulation, tolerance, stabilization, biomonitoring performance, engineered biosorption, and extractive removal represent distinct endpoints rather than interchangeable outcomes. High tissue metal concentration can indicate strong interception or retention, but it does not demonstrate remediation capacity unless biomass production, areal metal retention, metal mobility, harvestability, and functional persistence are evaluated. This distinction is consistent with phytoremediation theory and with bryophyte studies showing that tissue accumulation, metal sorption, moss bag monitoring, *in situ* stabilization, and field-scale removal have different mechanistic and operational meanings (Pulford and Watson, 2003; Ares et al., 2012; González and Pokrovsky, 2014; Wang et al., 2019; Tesser et al., 2021; Lu et al., 2022). Without clearer endpoint definition, bryophyte applications risk being either overstated as remediation technologies or undervalued as stabilizing and monitoring systems.

Microbiome- and omics-based research adds important explanatory potential, but it also illustrates the broader need for causal validation. Recent studies have expanded the range of candidate pathways and host-associated microbial patterns involved in stress responses, yet many remain descriptive and cannot determine whether observed changes improve host performance, reflect physiological injury, or arise from environmental filtering (Chen and Nelson, 2022; Boquete et al., 2022; Dangar et al., 2024; Ma et al., 2024; Zhu et al., 2024, Zhu et al., 2025). The same caution applies to microbiome-assisted tolerance, which remains less developed in bryophytes than in vascular plants. At the application level, slow growth and low biomass may further constrain the scalability of bryophyte-based remediation, even where local stabilization or sorption functions are promising (Tesser et al., 2021; Lu et al., 2022; Gezahegn et al., 2025; Singh and Choudhary, 2025). Progress will depend on linking candidate mechanisms to measurable ecological functions under field-relevant conditions.

### 7.3 Future directions

Future work should convert the proposed trait-informed framework into a quantitatively testable model. This will require standardized measurements of effective surface area, canopy architecture, wall-exchange capacity, metal localization, physiological injury, biomass, and recovery across representative life forms and lineages. Cross-species experiments should expose bryophytes to the same metal speciation, dose, duration, and hydration regime, allowing the contribution of individual traits to accumulation and tolerance to be estimated. Multi-omics analyses should be integrated with genetic manipulation, targeted biochemical validation, isotope or tracer approaches, and recovery experiments so that candidate pathways can be separated into causal mechanisms, compensatory responses, and consequences of damage.

Bridging laboratory and field scales will require mesocosm and reciprocal-transplant experiments that preserve realistic variation in substrate chemistry, climate, metal mixtures, and microbiomes. Biomonitoring studies should report the traits, preparation history, tissue age, exposure duration, and analytical procedures of the biological material, and should distinguish exposure indicators from effect indicators. Restoration studies should measure functional persistence, including cover maintenance, erosion control, metal stabilization, moisture regulation, and facilitation of later succession, rather than relying on tissue concentration alone. Controlled microbiome manipulation offers a promising direction, but microbiome-informed applications should be proposed only after beneficial effects are reproduced across host genotypes, environmental conditions, and contamination levels.

Overall, bryophytes should not be viewed merely as passive accumulators of pollutants. They are integrative biological systems in which environmental exposure, metal accumulation, tolerance mechanisms, microbial associations, and ecological function interact. Evidence for contrasting accumulation and tolerance strategies supports their use as biologically informative indicators of metal exposure (Boquete et al., 2021, Boquete et al., 2022). Field and experimental studies also support context-dependent roles in pioneer establishment, surface stabilization, moisture regulation, and reduced metal mobility in particular post-mining and tailing systems (Ren et al., 2021; Liao et al., 2024; Liu et al., 2024; Lin et al., 2024). These contributions should not be generalized as universal evidence of ecosystem recovery. Progress will depend on broader taxonomic coverage, stronger causal validation, standardized trait measurements, and explicit links between mechanistic responses and long-term field performance.
